# Oncological and Reproductive Outcomes in Patients With Advanced-Stage Ovarian Immature Teratoma: Experience From a Tertiary Center

**DOI:** 10.3389/fonc.2022.822341

**Published:** 2022-06-22

**Authors:** Dan Wang, Wei Cang, Shan Zhu, Congwei Jia, Dongyan Cao, Jiaxin Yang, Yang Xiang

**Affiliations:** ^1^ Department of Obstetrics and Gynecology, Peking Union Medical College Hospital, National Clinical Research Center for Obstetric and Gynecologic Diseases, Chinese Academy of Medical Sciences and Peking Union Medical College, Beijing, China; ^2^ Department of Pathology, Peking Union Medical College Hospital, Chinese Academy of Medical Science and Peking Union Medical College, Beijing, China

**Keywords:** immature teratoma, fertility preservation, advanced stage, oncological outcome, reproductive outcome

## Abstract

**Objective:**

To evaluate the oncological and reproductive outcomes in patients with advanced-stage ovarian immature teratoma (IMT).

**Methods:**

We retrospectively reviewed the medical records of patients with advanced-stage IMT who were treated with surgery between January 1985 and December 2020. Fertility-sparing surgery (FSS) was defined as preservation of the uterus and at least one adnexa. Oncological outcomes were compared between patients who underwent FSS and radical surgery. Patients who underwent FSS were also contacted to gather information about their menstrual history and reproductive outcomes.

**Results:**

Forty-six patients fulfilled the inclusion criteria, of whom 38 underwent FSS and eight were treated with radical surgery. Fifteen patients suffered recurrence after a median follow-up time of 74.2 months (range: 4.1–434.1 months). The 5-year disease-free survival (DFS) and overall survival (OS) rates were 69.1% and 89.9%, respectively. Multivariate analysis identified suboptimal cytoreductive surgery as the only independent risk factor for recurrence. There was no significant difference in DFS or OS between patients with different surgical procedures. Ten of the 15 relapsed patients had optimal salvage surgery and all remained alive with no evidence disease. Among the 32 patients who underwent FSS, 29 resumed menstruation after surgery, and five of seven patients who designed pregnancy achieved a total of five successful pregnancies.

**Conclusions:**

Ovarian IMT has a favorable prognosis, even when diagnosed at an advanced stage. FSS is feasible in patients with advanced-stage IMT who wish to preserve their fertility. Patients may benefit from optimal cytoreductive surgery during initial and salvage surgery.

## Introduction

Malignant ovarian germ cell tumors (MOGCTs) are rare cancers, accounting for <5% of all ovarian malignancies, with the highest rates in women aged 15–30 years (1, 2). MOGCTs comprise a heterogeneous group of tumors with different histological characteristics, including dysgerminoma, immature teratoma (IMT), yolk sac tumor, and mixed types (3). Ovarian IMTs represent about a third of all MOGCTs. Most IMTs are diagnosed at an early stage and occur in young patients, for whom fertility preservation is an important quality of life-related issue, and current guidelines suggest that fertility-sparing surgery (FSS) is feasible in young patients with early-stage IMT (4). However, 20%–30% of cases present with advanced-stage disease at diagnosis, and disease stage, histological type, and residual disease after surgery are important prognostic factors (1, 5).

MOGCTs show high chemosensitivity, and cytoreductive FSS has therefore also been applied in patients with advanced-stage MOGCTs (4). However, the rarity of MOGCTs means that the value of cytoreductive surgery remains unclear and less well-defined than in patients with ovarian epithelial cancer (6). The available evidence is derived mainly from small case series including women with heterogeneous neoplasms with variable biological behaviors (dysgerminoma, immature teratoma, yolk sac tumor, mixed types), or comprising a preponderance of cases with early-stage disease (7–9). It is therefore important to delineate the specific clinical behaviors of the different neoplasms. Furthermore, some previous studies did not include a control group of patients treated with radical surgery (10, 11). IMTs comprise a distinct subtype of MOGCTs consisting of tissues derived from three germ layers, with the grade determined by the quantity of immature neuroepithelium. Patients with IMT may relapse with mature elements (mature teratoma, gliomatosis peritonei) or experience malignant relapse (IMT) (12). Because most IMTs are diagnosed at an early stage, information on FSS in patients with advanced-stage IMT is limited. We report on the oncological and fertility outcomes in a relatively large series of young patients with advanced-stage IMT treated with FSS.

## Materials And Methods

We retrospectively reviewed patients with IMT treated at Peking Union Medical College Hospital (PUMCH) between January 1985 and December 2020, including patients referred for treatment after initial surgery elsewhere. The study was approved by the institutional review board of PUMCH (S-K1714). Information on patient demographics, surgical procedures, stage, histology, chemotherapy regimens, relapse characteristics, salvage treatment, and follow-up were collected from medical records. Disease stage was assessed according to the FIGO 2014 staging system for epithelial ovarian tumors, based on the intraoperative macroscopic description and by reviewing the pathological results. Patients with advanced-stage (II–IV) disease were selected for further analysis. The original histology slides were reviewed and confirmed by one of the authors (Congwei Jia) with experience in gynecological pathology. IMTs were graded according to the criteria developed by Norris (13) and modified by Scully and Robboy (14). Patients with mixed cell-type germ cell tumor after centralized histological review were excluded. Patients who were lost to follow-up were also excluded from the current study.

All patients underwent surgery as their initial treatment, consisting of either FSS (defined as preservation of the uterus and at least one adnexa) or radical surgery (defined as bilateral salpingo-oophorectomy with or without hysterectomy). Optimal debulking was defined as no residual tumor or residual disease ≤1 cm after surgery, and suboptimal debulking surgeries were defined as residual disease >1 cm. Adjuvant chemotherapy was given because of advanced stage, residual disease or high grade of teratoma.

Patients were followed-up every 3 months during the first 2 years, every 6 months during the following 3 years, and yearly thereafter. Follow-up included clinical examination and measurement of blood serum tumor markers (cancer antigen (CA)125, CA19-9, α-fetoprotein). Abdomen-pelvic computed tomography was decided by the physician overseeing the patient’s follow-up.

Patients with IMT may relapse with mature teratoma (growing teratoma syndrome, GTS) or IMT. Some patients have been reported in a previous study (15). Relapse was defined as the detection of disease at surgery performed for suspected recurrence. Only patients who relapsed with IMT (based on the final histological diagnosis) were enrolled in the analysis. Disease-free survival (DFS) was defined as time from the initial surgery to the date of recurrence or censoring. Overall survival (OS) was investigated as a secondary outcome and defined as time from initial surgery to the date of death or censoring. The associations between clinicopathological factors and recurrence were analyzed. Survival curves were calculated by the Kaplan–Meier method and compared with log-rank tests. A two-sided P-value < 0.05 was considered significant, and significant factors were included in further multivariate analysis. Hazard ratios were calculated for potential risk factors for relapse. All statistical analyses were performed using SPSS software (v. 20 s IBM Corp, Armonk, NY, USA).

## Results

### Clinicopathological Characteristics

**Figure 1:**
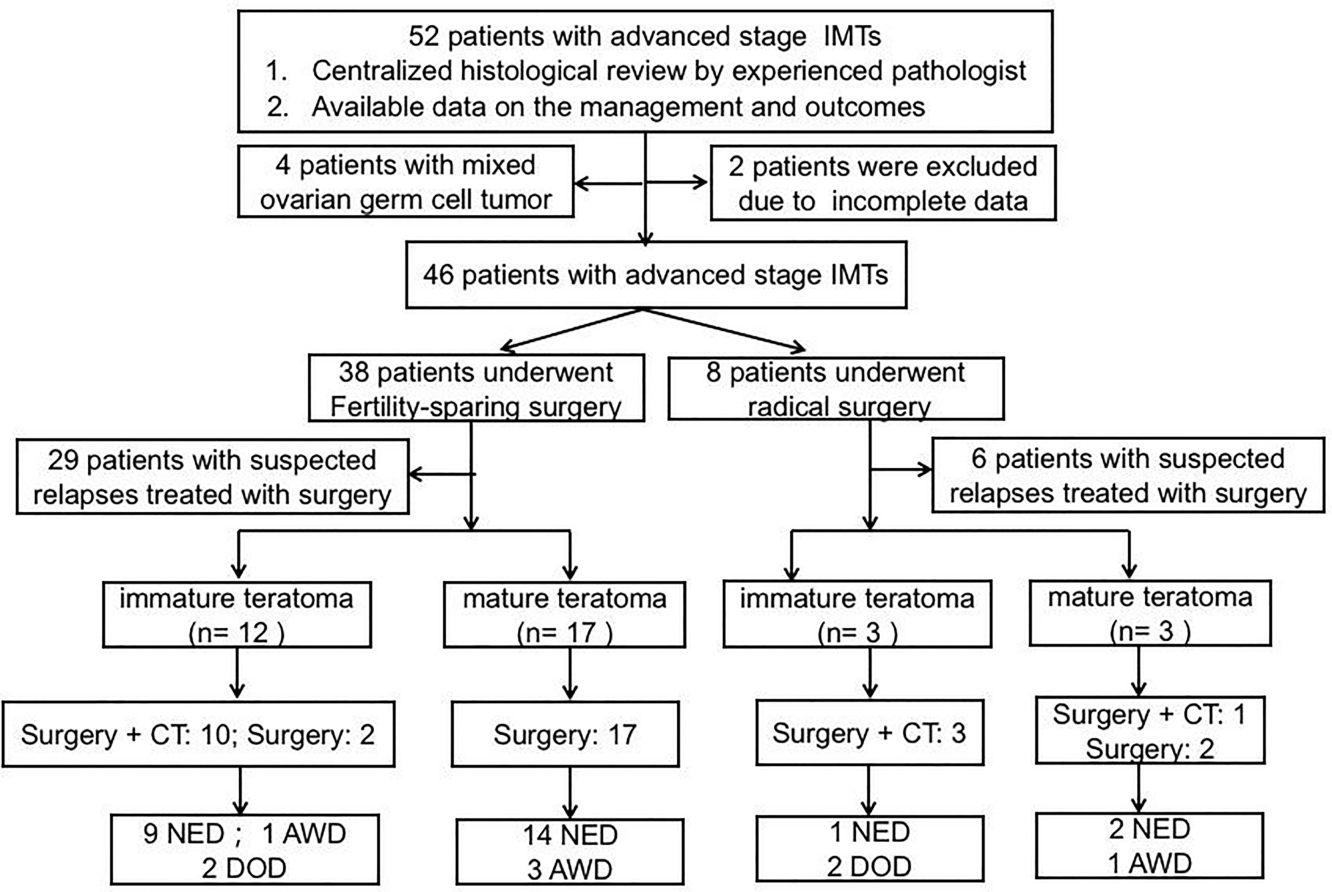
Flow chart summarizing management and outcomes in patients with advanced-stage IMT. AWD alive with disease, DOD died of disease, NED no evidence disease.

Fifty-two patients with advanced-stage IMT were treated at PUMCH between January 1985 and December 2020. The patient flow chart is presented in **Figure 1**. Four patients were excluded because they had mixed germ cell tumors after pathological review, and two patients were excluded because of a lack of follow-up information. A total of 46 patients thus fulfilled the inclusion criteria. Of these 46 patients, 16 received primary surgery at PUMCH and the remaining 30 patients were initially treated elsewhere and then transferred to our hospital for further management. These patients may have undergone a second, definitive surgical procedure after referral. Surgical extent was determined based on the initial and completion surgeries. The clinicopathological characteristics are presented in **Table 1**. The surgical procedures are described in **Table 2**. With regard to the status of residual disease, 20 patients received optimal cytoreduction (15 cases had no macroscopic residual tumor and 5 cases had residual disease ≤1 cm) while 26 patients had suboptimal cytoreduction (residual disease >1 cm). Among the 44 patients who received adjuvant chemotherapy after initial surgery, 29 received BEP (bleomycin, etoposide, cisplatin), 10 received BVP (bleomycin, vincristine, cisplatin), and five received other regimens.

**Table 1:** Clinical-pathological characteristics in patients with advanced-stage IMT.

Characteristics	
Age (median, years)	22 (6-39)
Nulliparous	35
Tumor size (median, cm)	20 (7-40)
FIGO stage	
II	16
III	30
Histological subtypes	
Pure immature teratoma	37
Immature teratoma with microscopic foci of yolk sac tumor	9
Tumor grade	
Grade 1	11
Grade 2-3	35
Gliomatosis Peritonei	14
Chemotherapy (types and number of cycles)	44
BEP regimen	29
2 cycles	2
3 cycles	3
4 cycles	15
Others	8
BEP followed by EP regimen (5 cycles)	1
BVP regimen	10
1 cycle	1
3 cycles	2
4 cycles	1
6 cycles	4
Others	2
Other regimens	5
Oncological outcomes	
IMTs	15
GTS	20
Death	4

FIGO, the International Federation of Obstetrics and Gynecology; GTS, growing teratoma syndrome; IMT, immature teratoma; BEP, bleomycin, etoposide, cisplatin; BVP, bleomycin, vincristine, cisplatin; EP, etoposide, cisplatin.

**Table 2:** Characteristics of patients at the management of advanced-stage IMT.

Characteristics	N = 46
Surgical procedure	
Fertility-sparing surgery	38
Radical surgery	8
Surgical procedures for debulking surgery	
Ovarian procedures	46
Ovarian cystectomy	4
Unilateral salpingo-oophorectomy	34
Hysterectomy and bilateral salpingo-oophorectomy	8
Lymphadenectomy (pelvic and/or para-aortic)	16
Complete omentectomy	37
Large peritonectomies	38
Appendectomy	10
Small bowel resection	1
Surgical outcomes	
No macroscopic residual tumor	15
Residual disease ≤1cm	5
Residual disease >1 cm	26

IMT, immature teratoma.

### Oncological Outcomes

A total of 35 patients underwent surgeries for suspected relapse after a median follow-up time of 74.2 months (range: 4.1–434.1 months). The management and outcomes of the included patients are summarized in **Figure 1**. Fifteen patients had recurrent disease in the form of IMT. The specific distributions of the relapses were as follows: pelvis (n=6), abdomen (n=1), pelvis + abdomen (n=7), and abdomen + lung (n=1). Thirteen patients underwent secondary debulking surgery plus chemotherapy and two underwent surgical cytoreduction alone. Ten patients had optimal cytoreductive surgery, all of whom remained alive with no evidence disease. Among the other five patients who received suboptimal surgery, one remained alive with disease and the other four patients had died of disease progression by the end of the last follow-up.

Twenty patients developed recurrence in the form of GTS, all of whom underwent secondary cytoreductive surgery. One patient received adjuvant chemotherapy because the histological report revealed a mature teratoma with some carcinoid tumor components (5%). The distributions of the relapses were as follows: pelvis (n=2), abdomen (n= 4), and pelvis + abdomen (n=14). Optimal salvage surgery was performed in 16 patients, and none experienced GTS recurrence. All these patients remained alive at the end of follow-up, with no evidence of disease. The remaining four patients underwent incomplete resection, and all were alive with disease at the end of follow-up.

**Figure 2:**
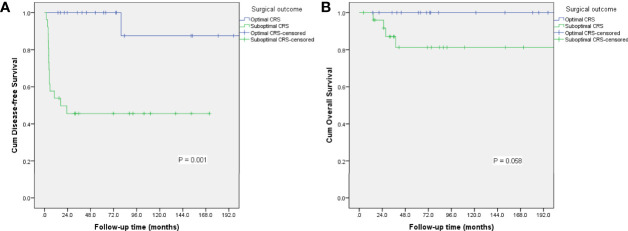
Survival curves for disease-free survival **(A)** and overall survival **(B)** according to surgical outcome.

The 5-year disease-free survival (DFS) and overall survival (OS) rates in whole study group were 69.1% and 89.9%, respectively. We analyzed potential associations between clinicopathological factors and recurrence (**Table 3**). DFS was significantly associated with surgical outcome and initial treatment site in univariate analysis (P = 0.001, 0.031, respectively), but only surgical outcome was identified as an independent risk factor for relapse in multivariate analysis (hazard ratio = 11.831, 95% confidence interval: 1.409–99.338, P = 0.023). The 5-year DFS and OS rates in patients with FSS were 70.3% and 93.8%, respectively, and the equivalent rates in patients with radical surgery were 62.5% and 72.9%, respectively. Surgical procedure (FSS vs radical surgery) was not significantly associated with DFS or OS. The DFS and OS curves according to surgical outcomes are presented in **Figure 2**. Parity, FIGO stage, tumor rupture, lymphadenectomy, histology, tumor grade, and gliomatosis peritonei were not associated with recurrence.

**Table 3:** Risk factors for recurrence in patients with advanced-stage IMT.

Factors	Univariate analysis			Multivariate analysis	
	5-year DFS (%)	HR (95% CI)	*P*	HR (95% CI)	*P*
Parity			0.681		
Nulliparous	67.8	1			
Parous	72.7	0.767 (0.215-2.736)			
Initial treatment			0.031		0.519
Our clinic	92.9	1		1	
Outside facility	56.5	4.479 (1.009-19.887)		1.675 (0.350-8.021)	
FIGO stage			0.346		
II	75.0	1			
III	65.9	1.723 (0.548-5.420)			
Tumor rupture			0.747		
No	68.9	1			
Yes	69.2	1.185 (0.421-3.336)			
Surgical procedures			0.741		
Radical surgery	62.5	1			
FSS	70.3	0.808 (0.228-2.866)			
Surgical outcomes			0.001		0.023
Optimal CRS	100	1		1	
Suboptimal CRS	45.6	14.836 (1.946-113.092)		11.831 (1.409-99.338)	
Lymphadenectomy			0.118		
Yes	87.5	1			
No	59.0	2.654 (0.742-9.492)			
Histology			0.914		
Pure IMTs	69.9	1			
IMTs with microscopic foci of YST	66.7	0.933 (0.263-3.311)			
Tumor grade			0.792		
G1	62.3	1			
G2-G3	71.1	0.857 (0.273-2.695)			
GP at initial surgery			0.111		
No	62.2	1			
Yes	84.4	0.317 (0.071-1.411)			

CI, confidential interval; CRS, cytoreductive surgery; DFS, disease-free survival; FIGO, the International Federation of Obstetrics and Gynecology; FSS, Fertility-sparing surgery; GP, gliomatosis peritonei; GTS, growing teratoma syndrome; HR: hazard ratio; IMT, immature teratoma; YST, yolk sac tumor.

### Reproductive Outcomes

Among 38 patients who received FSS, two patients died due to disease progression and four patients underwent hysterectomy after relapse. Thirty-two patients were therefore available to evaluate menstruation. Of these, 29 patients reported regular menstruation during follow-up. The other three did not have regular menses following treatment: one patient remained premenarchal, another patient had secondary amenorrhea due to bilateral salpingo-oophorectomy, and the third patient had oligomenorrhea. Five of the seven patients who desired fertility achieved singleton pregnancies (one each, including one preterm pregnancy). All these patients received prior platinum-based chemotherapy, and no congenital anomalies were observed in the babies.

## Discussion

The result of the current study suggests that FSS was associated with excellent oncological outcomes in patients with advanced-stage IMT, with most IMT recurrences successfully salvaged by secondary surgery. The 5-year DFS and OS rates in patients treated with FSS were 70.3% and 93.8%, respectively. Furthermore, more than half (17/29) of the patients relapsed with GTS which was previously reported to have an excellent prognosis (15, 16). According to the European Society of Medical Oncology guidelines, FSS is not contraindicated in patients with MOGCTs who want to preserve their fertility, even among patients with advanced-stage disease (4). However, the rarity of advanced-stage MOGCTs means that most reports of FSS in these patients have included a heterogeneous group of patients with tumors with variable biological behaviors and prognoses, or studies that lacked a control group of patients with radical surgery, making it difficult to draw specific conclusions about the use of FSS for advanced-stage IMT (10, 11). Low et al. reported an excellent OS rate (94.4%) among 18 patients with advanced-stage MOGCTs managed with FSS and adjuvant chemotherapy (7), and Park et al. reported 5-year DFS and OS rates of 89% and 91%, respectively, in 46 patients with stage II–IV MOGCT treated with FSS (10). Tamauchi et al. reported reproductive outcomes in 105 patients with MOGCTs. Among them, 8 of 40 patients with successful pregnancies had FIGO stage II or III disease (17). They accordingly concluded that FSS could be performed safely in young patients with advanced-stage MOGCT. Nasioudis et al. recently evaluated the safety of uterine preservation in premenopausal women with advanced-stage MOGCT using data from the National Cancer Data Base (NCDB). The rate of uterus preservation among 526 patients was 79.8%, with no impact on survival (18). However, no specific information on IMT was provided, and differences in progression-free survival could not be analyzed because of a lack of information about tumor recurrence in the NCDB. The current series thus provides a meaningful sample of patients with advanced-stage IMT treated with FSS or radical surgery, including complete reports of oncological and reproductive outcomes. Our result indicated that FSS did not have a negative effect on DFS or OS in patients with advanced-stage IMT, suggesting that FSS was a feasible option for young patients with advanced-stage IMT wishing to preserve their fertility.

The principle of primary cytoreductive surgery for advanced MOGCT is derived from the use of debulking surgery for ovarian epithelial cancer, defined as resection of as much gross tumor as possible (1). Given the rarity of MOGCTs, the role of aggressive surgery for patients with advanced disease is not well established. However, some retrospective studies demonstrated a benefit of minimal residual disease at completion of primary cytoreductive surgery (8, 19). In a Gynecologic Oncology Group study, 68% pf patients who underwent suboptimal surgery had disease progression compared with only 28% of patients who underwent complete cytoreduction (19). Park et al. identified residual disease as an independent risk factor for reduced DFS and OS (10). Karalok et al. recently evaluated the survival effect of cytoreductive surgery in 31 patients with advanced-stage MOGCT, and found that optimal or maximal debulking surgery significantly improved DFS and OS (8). In the current study, the 5-year DFS in patients with suboptimal surgery was only 45.6% compared with 100% in patients with optimal cytoreduction (P = 0.001). Surgical outcome was identified as an independent risk factor for relapse in these patients. Among the 20 patients who received optimal cytoreductive surgery, only one patient suffered from recurrence and was successfully salvaged by secondary surgery, while 14 of 26 patients with suboptimal surgery relapsed with IMT. Maximal resection of all gross disease should thus be the aim in patients with advanced IMT, to increase the chance of survival.

Although most patients with MOGCT can be cured, around 15%–20% with advanced-stage disease at presentation experience relapse and need salvage treatment, usually within 24 months after their initial treatment (20). The value of salvage treatment in patients with relapsed testicular germ cell tumors is well defined (21), while its value in female counterparts is less clear (22), and many clinicians thus approach the issue in a similar way to that for testicular tumors (23). However, data are limited to small retrospective studies. Wu et al. reported their experience of the role of salvage surgery in 34 patients with chemorefractory MOGCT, and found a 3-year survival rate of 80% in patients with optimal cytoreductive surgery compared with only 21.05% in those with suboptimal surgery (24). They concluded that optimal salvage surgery should be recommended in patients with chemorefractory MOGCT. Lai et al. noted that residual tumor ≥1 cm after salvage treatment was significantly associated with death. They also found that patients with IMT or dysgerminoma had a better prognosis compared with other types of MOGCT (22). Munkarah and associates reported the oncological outcomes of salvage surgery in 20 patients with MOGCT who failed primary treatment. All patients with IMT remained alive after salvage surgery, except for one who was alive with disease. They noted that patients with IMT who underwent second cytoreductive surgery had significantly better survival than patients with other types of MOGCT (25). However, this series only included eight cases of IMT. Notably, IMT is a subtype of MOGCT in which secondary cytoreduction may play a strong role. In one review, Wu et al. summarized 11 articles to evaluate the role of secondary debulking surgery in patients with recurrent IMT. A total of 27 patients with IMT who underwent salvage surgery after primary treatment failure were available, of whom 23 were alive at the end of follow-up (26). It is therefore possible that salvage surgery may be beneficial in patients with IMT. In the current study, 15 patients relapsed with IMT, including 10 who underwent optimal salvage surgery and five who did not. By the end of follow-up, all patients with optimal secondary surgery were alive, while four of the five with suboptimal surgery died of the disease. These results suggest that the status of salvage surgery was associated with OS. Optimal surgical debulking is thus critical at the time of diagnosis and in a recurrence setting, and surgeons should aim to remove recurrent IMTs completely. Residual disease after surgery for GTS also represents a risk factor for GTS recurrence in our previous report (15), and optimal cytoreduction is also required to remove the compression symptoms related to bulky benign disease and to reduce GTS recurrence.

IMT shows distinct biological behavior compared with other subtypes of MOGCTs. It may relapse with pure IMT or mature teratoma defined as GTS (12). Due to its rarity, GTS may be misdiagnosed as disease progression or recurrence (27). However, it is important to differentiate between IMT relapse and GTS, because both the treatment strategy and prognosis differ. Patients with IMT relapse require complete resection with adjuvant chemotherapy, while the management of GTS mainly involves complete resection of all macroscopic disease to relieve compression syndrome, with no need for adjuvant chemotherapy, because GTS is potentially chemoresistant. In the current series, 35 patients had suspected relapse. After surgery, 15 of these were confirmed with IMT and the remaining 20 with mature teratomas. Surgery should be performed to evaluate the nature of the relapse, as either IMT requiring further adjuvant chemotherapy, or mature elements needing no further management. This would help to exclude active residual tumors and prevent GTS, with the rare possibility of dedifferentiation of the teratoma into ovarian malignancies (23).

This study had some limitations. First, it was a retrospective study conducted in a tertiary center and may thus have had referral bias, given that most of the patients were initially diagnosed elsewhere and then transferred to our hospital for further management. Most patients seeking further treatment in our hospital already had unfavorable factors such as residual disease, which may have led to over-interpretation of the results. Second, the relatively low number of cases in the current study may limit the generalizability of the results. However, our study also had some advantages. First, it included one of the largest series in the published literature involving only patients with advanced-stage IMT, focusing specifically on the outcomes of FSS, and thus represents a valuable contribution to the limited body of knowledge on this topic. Second, all our cases underwent central pathological review by an experienced gynecological pathologist. Previous studies included heterogeneous patients with different recurrence risks, while the inclusion of only patients with IMT meant that the current study was conducted in a homogeneous population. Finally, we reported the obstetric outcomes of patients with advanced-stage disease after FSS, which have rarely been detailed in previous reports.

In conclusion, patients with ovarian IMT have a favorable prognosis, even when diagnosed at an advanced stage. FSS is not associated with increased recurrence compared with radical surgery, suggesting that FSS is a feasible option in patients with advanced-stage IMT who wish to preserve their fertility. Suboptimal surgical outcome is significantly associated with relapse in these patients, but most relapses can be successfully salvaged by optimal secondary cytoreductive surgery. IMTs comprise a specific group of MOGCTs, for which secondary cytoreductive surgery may play a strong role. Due to the limited number of cases of advanced-stage IMT, future collaborative studies with pooled data are needed to confirm these conclusions.

## Data Availability Statement

The raw data supporting the conclusions of this article will be made available by the authors, without undue reservation.

## Ethics Statement

The studies involving human participants were reviewed and approved by the institutional review board of Peking Union Medical College Hospital (S-K1714). Written informed consent to participate in this study was provided by the participants’ legal guardian/next of kin.

## Author Contributions

Conception and design: DW, CJ, DC, and YX; Acquisition of data: DW, WC, SZ, CJ, DC, JY, and YX; Analysis and interpretation of data: DW, WC, SZ, CJ, DC, JY, and YX; Manuscript writing: DW, WC and YX; Funding acquisition: YX; Critical review of the manuscript: DW, WC, SZ, CJ, DC, JY, and YX; Final approval of manuscript: DW, WC, SZ, CJ, DC, JY, and YX; All authors contributed to the article and approved the submitted version.

## Funding

This work was supported by grants from the National Natural Science Foundation of China (No. 81971475).

## Conflict of Interest

The authors declare that the research was conducted in the absence of any commercial or financial relationships that could be construed as a potential conflict of interest.

## Publisher’s Note

All claims expressed in this article are solely those of the authors and do not necessarily represent those of their affiliated organizations, or those of the publisher, the editors and the reviewers. Any product that may be evaluated in this article, or claim that may be made by its manufacturer, is not guaranteed or endorsed by the publisher.
